# Supplementary material for: Amyloid formation and depolymerization of tumor suppressor p16INK4a are regulated by a thiol-dependent redox mechanism
Source: Nat Commun. 2024 Jul 1;15:5535. doi: 10.1038/s41467-024-49581-7 (PMC11217399; doi:10.1038/s41467-024-49581-7)
Supplement: Supplementary file 1 — Supplementary Information [file 41467_2024_49581_MOESM1_ESM.pdf]

Supplementary Materials for

Amyloid formation and depolymerization of tumor suppressor p16^{INK4a} are regulated by a thiol-dependent redox mechanism

Sarah G. Heath[1], Shelby G. Gray[2], Emilie M. Hamzah[2], Karina M. O'Connor[1], Stephanie M. Bozonet[1], Alex D. Botha[1], Pierre de Cordovez[1], Nicholas J. Magon[1], Jennifer D. Naughton[1], Dylan L. W. Goldsmith[2], Abigail J. Schwartfeger[2], Margaret Sunde[3], Alexander K. Buell[4], Vanessa K. Morris[2,5]*, Christoph Göbl[1,5]*

*** Corresponding authors.** Emails: vanessa.morris@canterbury.ac.nz, christoph.goebl@otago.ac.nz

This PDF file includes:

Supplementary Figures 1-9

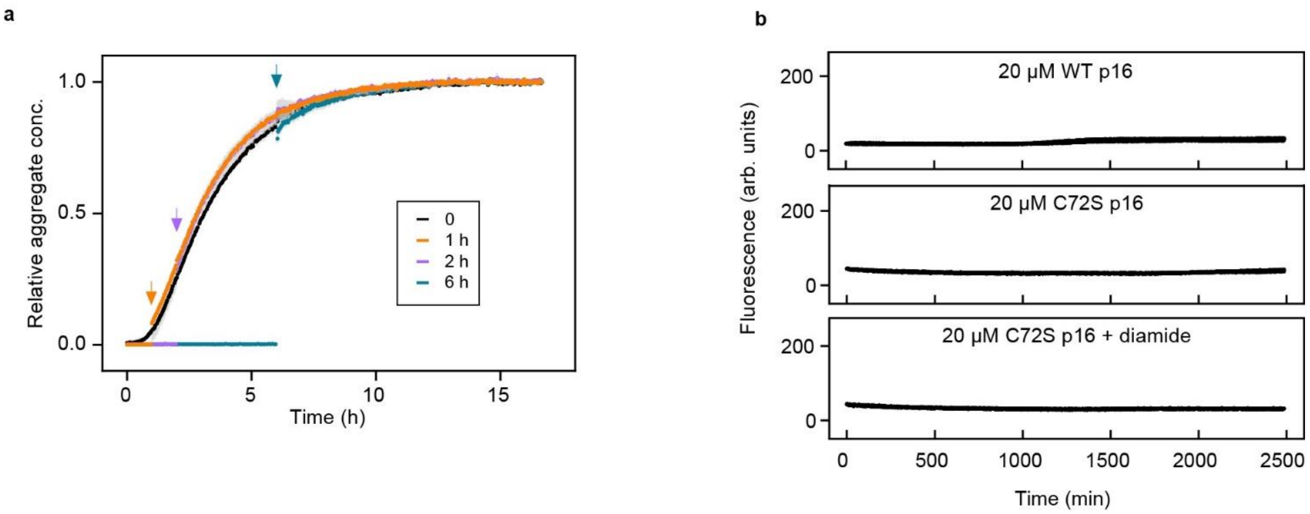

Supplementary Fig. 1: (**a**) Addition of ThT dye does not impact the rate of p16 amyloid formation. The assay (n=4) was conducted under standard conditions and ThT was added at time zero or 1, 2 or 6 h after assay initiation. Gray error bars represent standard deviation. (**b**) ThT fluorescence analysis of wild-type and C72S p16. Assays were conducted under standard conditions using 20 µM untreated wild-type protein, 20 µM untreated C72S protein, or 20 µM C72S protein treated with 200 µM diamide. Data are plotted from three experiments.

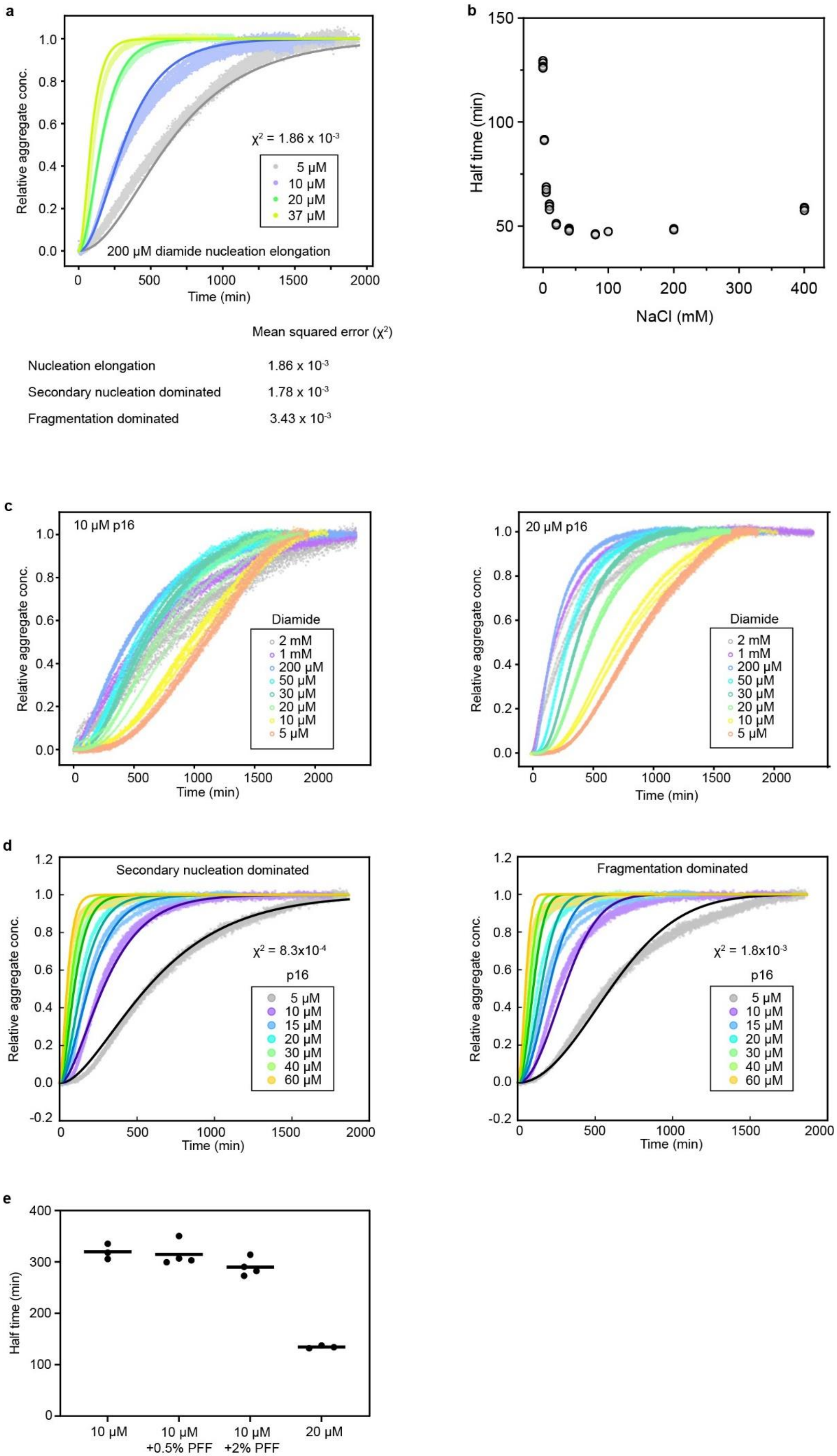
a
Relative aggregate conc.
Time (min)
χ² = 1.86 x 10⁻³
5 μM
10 μM
20 μM
37 μM
200 μM diamide nucleation elongation
Mean squared error (χ²)
Nucleation elongation 1.86 x 10⁻³
Secondary nucleation dominated 1.78 x 10⁻³
Fragmentation dominated 3.43 x 10⁻³
b
Half time (min)
NaCl (mM)
c
10 μM p16
20 μM p16
Diamide
2 mM
1 mM
200 μM
50 μM
30 μM
20 μM
10 μM
5 μM
d
Secondary nucleation dominated
χ² = 8.3x10⁻⁴
Fragmentation dominated
χ² = 1.8x10⁻³
p16
5 μM
10 μM
15 μM
20 μM
30 μM
40 μM
60 μM
e
Half time (min)
10 μM
10 μM +0.5% PFF
10 μM +2% PFF
20 μM

Supplementary Fig. 2: (**a**) ThT fluorescence analysis of 5–37 μM p16 oxidized with 200 μM diamide. Symbols represent data from four measurements with solid lines showing the nucleated elongation polymerisation fit (AmyloFit webserver). Below, the mean squared error values resulting from fitting this data set with the secondary nucleation dominated and fragmentation dominated mechanisms. (**b**) p16 aggregation half times as a function of NaCl concentration from fig. 1**i**. The half times plotted result from four measurements per NaCl concentration. (**c**) ThT-monitored aggregation kinetics of 10 μM and 20 μM p16 treated with 5 μM–2 mM diamide. Data is presented from four measurements. (**d**) Aggregation kinetics of p16 as a function of monomer concentration monitored by ThT fluorescence. Aggregation of monomer p16 was initiated at t0 by addition of diamide at a 1:10 molar ratio with respect to protein monomer concentration. Assays were conducted under standard conditions and symbols represent data from four measurements with solid lines showing the secondary nucleation dominated (global fitting parameters $k_+k_n = 6.59 \times 10^8$ M^{-nc} h^{-2}, $k_+k_2 = 5.83 \times 10^3$ M^{-n2-1} h^{-2}) and fragmentation dominated fits (global fitting parameter $k_+k_n = 2.81 \times 10^8$ M^{-nc} h^{-2}, $k_+k_- = 4.98 \times 10^{-6}$ M^{-1} h^{-2}) (AmyloFit webserver). (**e**) Half times of aggregation kinetic data in Fig. 1**h**. Horizontal lines represent the mean from four measurements.

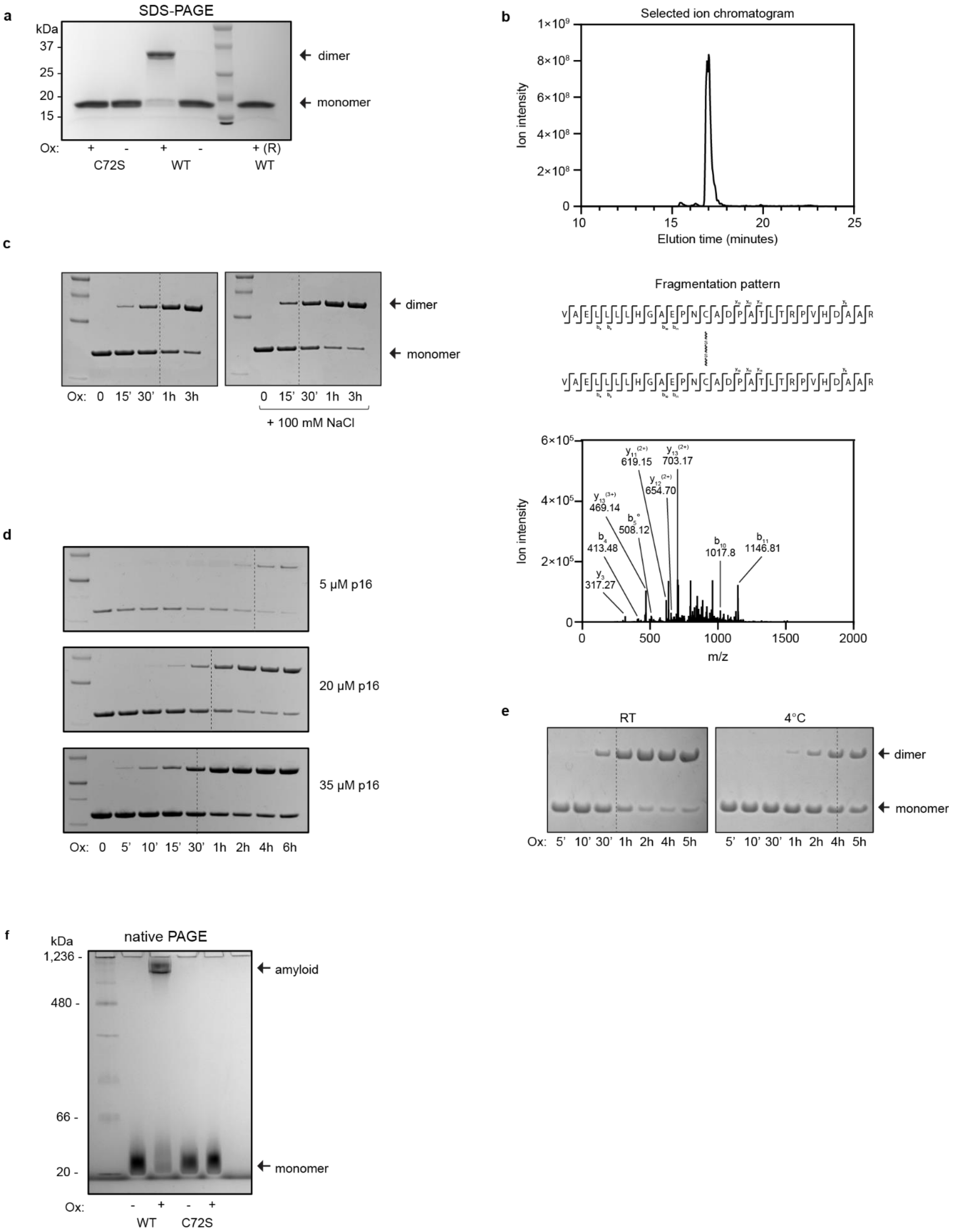
a
SDS-PAGE
kDa
37
25
20
15
dimer
monomer
Ox: + - + - + (R)
C72S WT WT
b
Selected ion chromatogram
1×10^9
8×10^8
6×10^8
4×10^8
2×10^8
0
Ion intensity
10 15 20 25
Elution time (minutes)
Fragmentation pattern
VAELLLLHGAEPNCADPATLTRPVHDAAR
VAELLLLHGAEPNCADPATLTRPVHDAAR
6×10^5
4×10^5
2×10^5
0
Ion intensity
$y_{11}^{(2+)}$ 619.15
$y_{13}^{(2+)}$ 703.17
$y_{12}^{(2+)}$ 654.70
$y_{13}^{(3+)}$ 469.14
$b_5°$ 508.12
b_4 413.48
y_3 317.27
b_{10} 1017.8
b_{11} 1146.81
0 500 1000 1500 2000
m/z
c
dimer
monomer
Ox: 0 15' 30' 1h 3h
0 15' 30' 1h 3h
+ 100 mM NaCl
d
5 µM p16
20 µM p16
35 µM p16
Ox: 0 5' 10' 15' 30' 1h 2h 4h 6h
e
RT
4°C
dimer
monomer
Ox: 5' 10' 30' 1h 2h 4h 5h
5' 10' 30' 1h 2h 4h 5h
f
native PAGE
kDa
1,236 -
480 -
66 -
20 -
amyloid
monomer
Ox: - + - +
WT C72S

Supplementary Fig. 3: (**a**) Oxidation of wildtype p16 promotes dimerization, as shown by the denaturating conditions of non-reducing SDS-PAGE. Plus and minus indicate presence or absence of oxidant (200 µM diamide) for 24 h. Reduction with 10 mM DTT, indicated by (R), of oxidized WT p16 abolishes the mass shift. Oxidant treatment does not affect migration of C72S p16. (**b**) Selected ion chromatogram for p16 disulfide (VAELLLLHGAEPNCADPATLTRPVHDAAR-VAELLLLHGAEPNCADPATLTRPVHDAAR) (m/z (charge state): 611.19 (+10), 678.99 (+9), 763.74 (+8), 872.70 (+7), 1017.99 (+6), 1221.38 (+5), 1526.48 (+4)). Fragmentation pattern of p16 disulfide (763.86 m/z (+8), 16.8 min). The loss of water from a fragment ion is denoted by '°' where y_x° is y_x-H_2O. Source data are provided as a Source Data file. (**c**-**e**) Non-reducing SDS-PAGE analysis showing dependence of the rate of 20 µM p16 dimerisation on buffer salt concentration (**c**), initial p16 monomer concentration (**d**), and temperature (**e**). Dotted lines guide the eye to the approximate half time of dimerization. (**f**) Native PAGE analysis shows 200 µM diamide treatment of wild-type p16 for 24h triggers a several-fold mass shift. Oxidation of C72S p16 does not affect migration indicating a dependence on C72 for the mass shift.

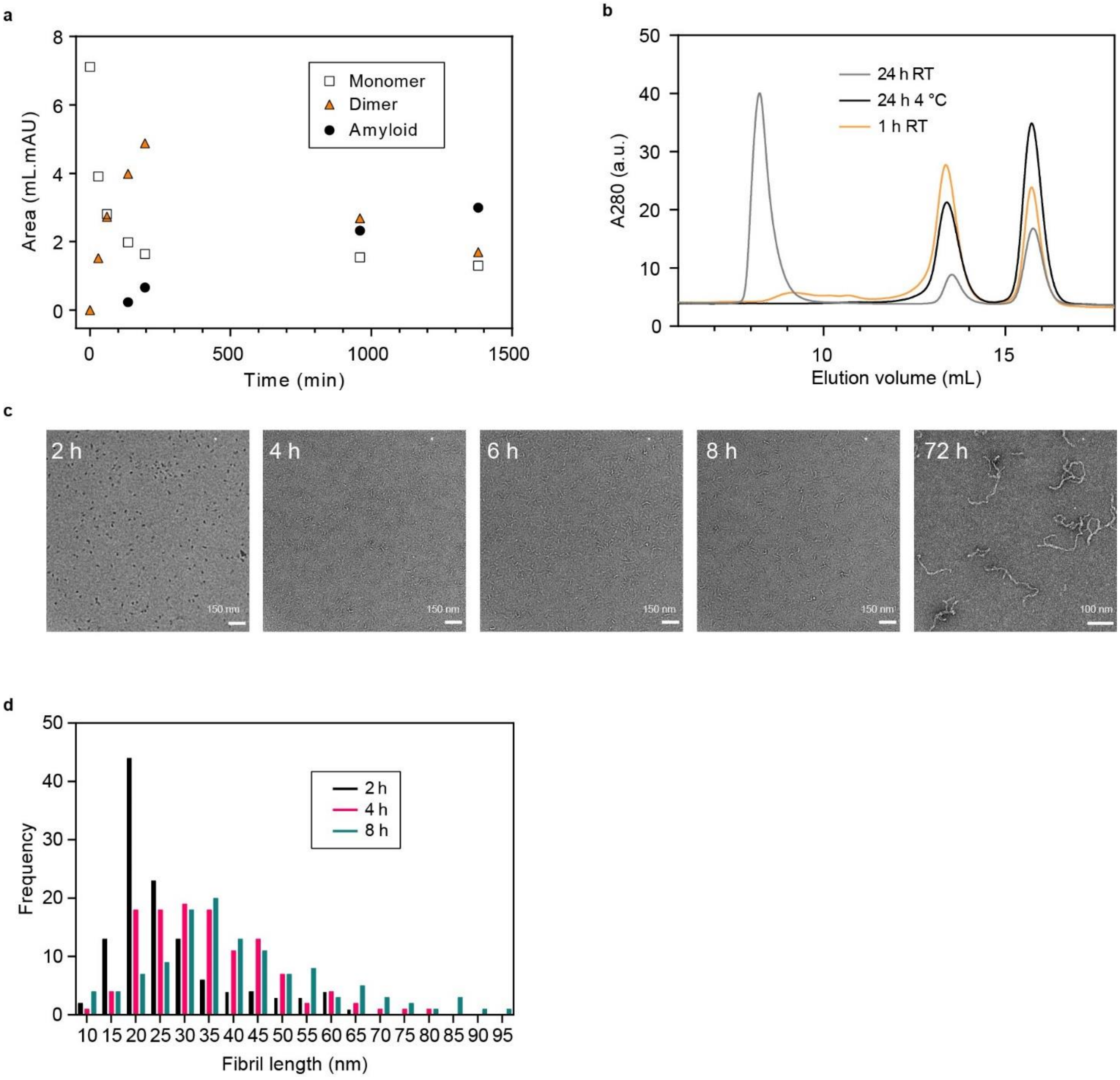

Supplementary Fig. 4: (**a**) Quantification of monomer, dimer and amyloid peaks from size exclusion chromatography elution profiles (data from Fig. 2b). (**b**) Size exclusion chromatography elution profiles comparing p16 aggregation kinetics at 4 °C and room temperature (RT). SEC running buffer includes 40 mM NaCl for improved resolution. (**c**) Negative-stained transmission electron micrographs of 20 µM p16 treated with 200 µM diamide after 2-72 h oxidation. (**d**) Distribution of fibril lengths of the 2, 4 and 8 h electron micrographs in (**c**).

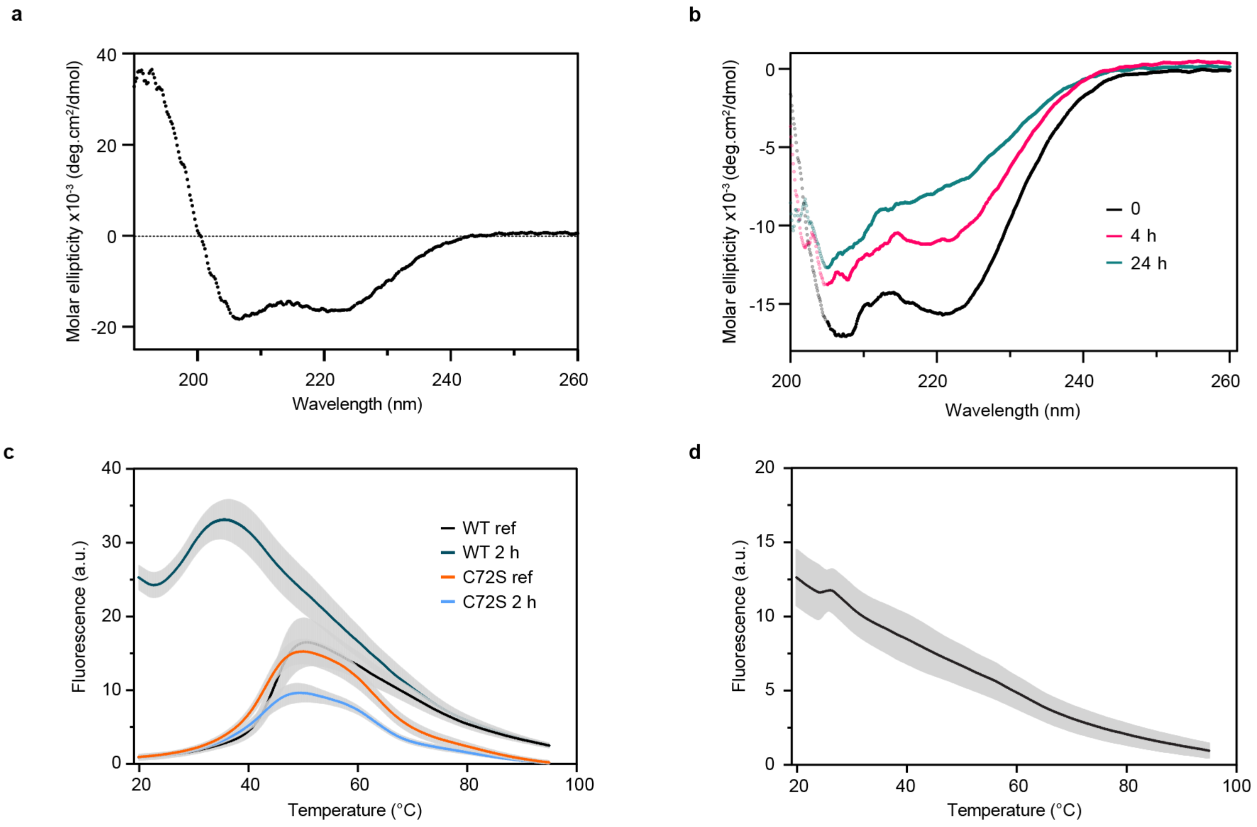

Supplementary Fig. 5: (**a**) Reference circular dichroism spectrum of untreated monomeric 5 µM p16 in 10 mM phosphate buffer, pH 7.4. Presented data is buffer subtracted but no smoothing functions have been applied. (**b**) Circular dichroism spectroscopy analysis of 20 µM p16 secondary structure following oxidation with 200 µM diamide under standard conditions (4 mM HEPES, pH 7.4). Full scans at 4 h and 24 h show a loss of α-helical signal following oxidation. (**c**) Differential scanning fluorimetry analysis of 20 µM WT and C72S p16 samples untreated (ref) or oxidized with 200 µM diamide for 2 h. Error bars represent standard deviation from five measurements. (**d**) Differential scanning fluorimetry analysis of 20 µM WT p16 after 24 h of oxidation with 200 µM diamide. Error bars represent standard deviation from five measurements.

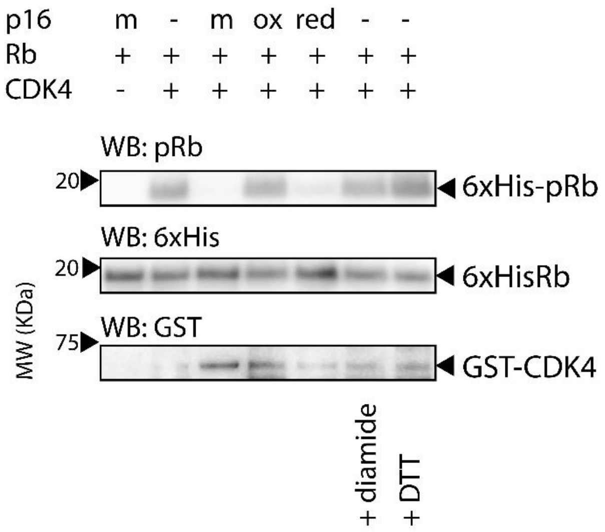

Supplementary Fig. 6: Kinase assay of CDK4/Cyclin D1, Rb and ATP in the presence of 2-fold excess of monomeric p16 (m), amyloid p16 (ox) and reduced amyloids (red). Oxidation and reduction have been performed for 24 h at room temperature. The last two lanes show results of experiments that did not contain p16 but included the oxidant (diamide) or reductant (DTT) as a control. Blots are representative of two independent experiments.

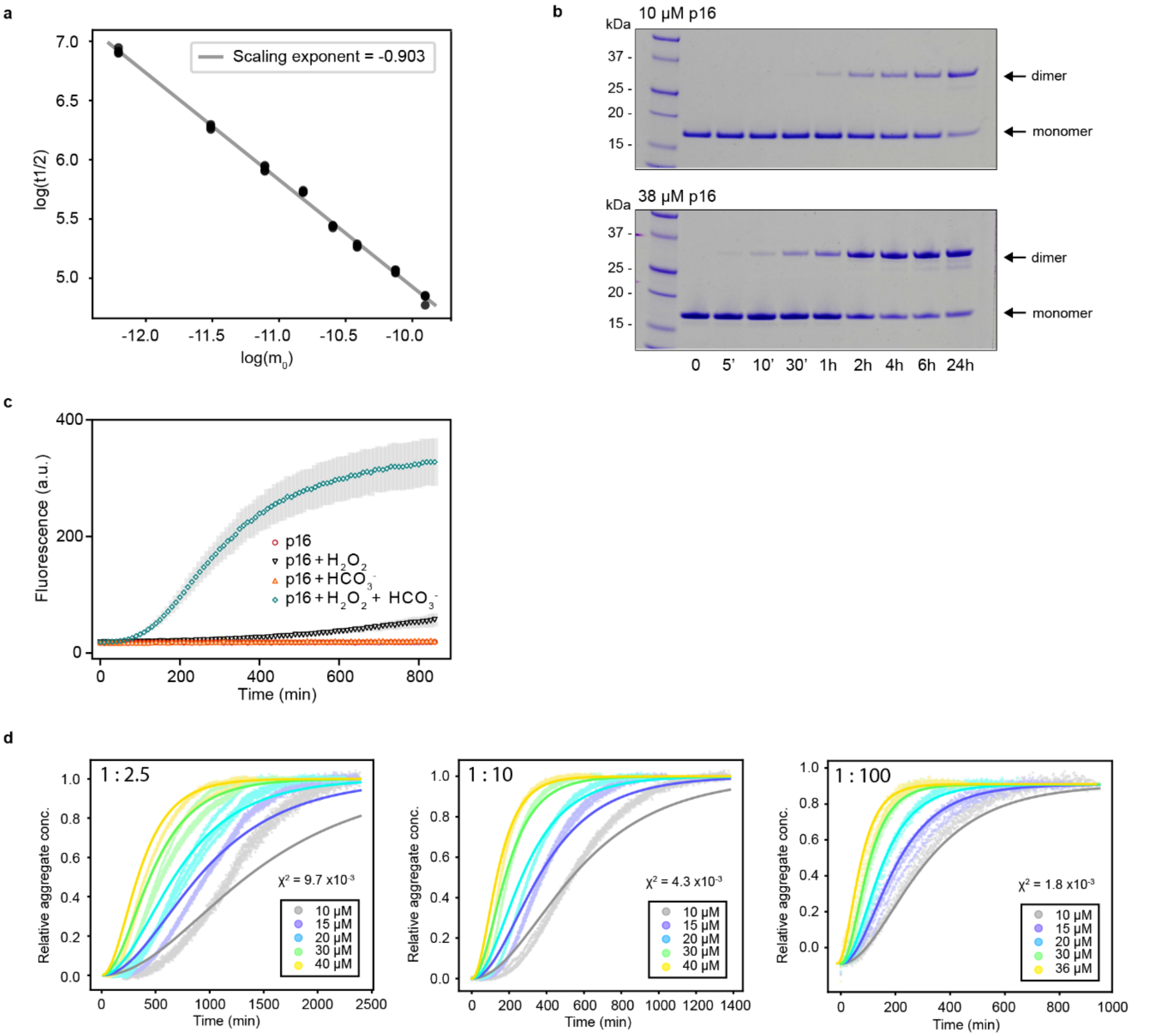

Supplementary Fig. 7: (**a**) Double logarithmic plot of aggregation half times (t½) as a function of p16 monomer concentration (m_0). Aggregation of monomer p16 was initiated at t_0 by addition of H_2O_2 at a 1:10 molar ratio with respect to monomer concentration. The assay was conducted in 4 mM HEPES buffer at pH 7.4 with 25 mM bicarbonate at 25 °C. A linear fit between logarithmic half-times and protein concentrations yields a slope of -0.903 and suggests that the dominant mechanism of amyloid formation is unchanged across the concentration range. (**b**) Rate of p16 dimerization measured by non-reducing SDS-PAGE. 10 and 38 µM p16 were oxidized with a 10-fold molar excess of H_2O_2 in the presence of 25 mM bicarbonate buffer pH 7.4. After the specified treatment time 10 mM NEM was added to prevent further dimerization of remaining monomeric p16. (**c**) The bicarbonate buffer requires H_2O_2 to trigger p16 amyloid formation. In the absence of H_2O_2 (orange triangles), p16 does not aggregate in 25 mM bicarbonate buffer, as measured by ThT fluorescence (data overlapping with p16 negative control). Error bars represent the standard deviation from four measurements. (**d**) Increasing the molar ratio of oxidant to p16 improves the fit to the nucleation elongation model of amyloid formation (AmyloFit). Reactions were conducted in the presence of 4 mM HEPES, 25 mM bicarbonate buffer pH 7.4, and the ratio between protein and oxidant is displayed.

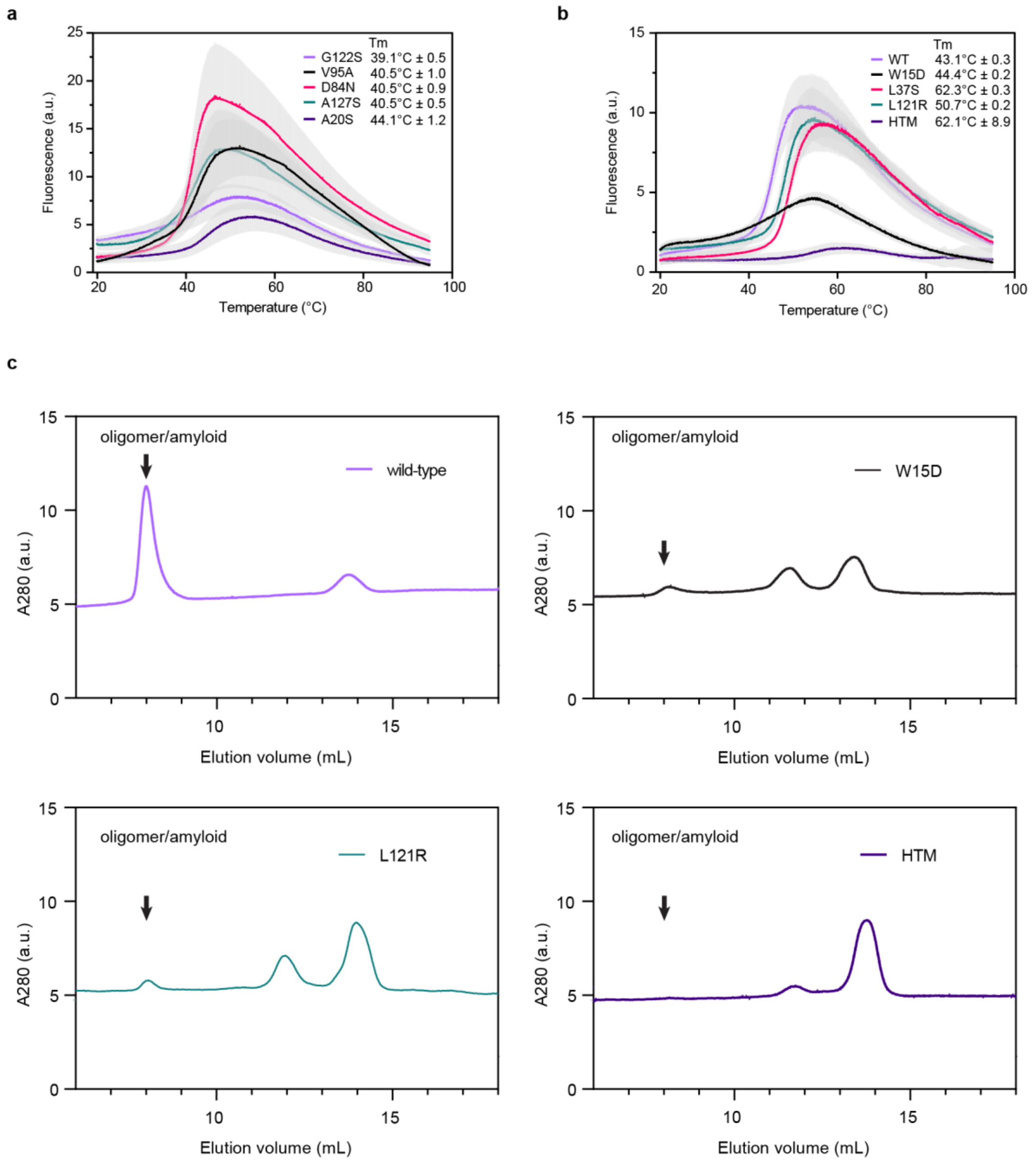

Supplementary Fig. 8: (**a**) Measurement of melting temperatures of several cancer-derived mutations using differential scanning fluorimetry. Melting temperatures of the stabilizing mutations can be found in (**b**). (**c**) Size exclusion chromatography of 20 μM protein samples after 24h of oxidation with 200 uM diamide. The formation of oligomers or fibrils is greatly decreased in stabilizing mutant samples, their position is indicated by the wild-type reference and an arrow.

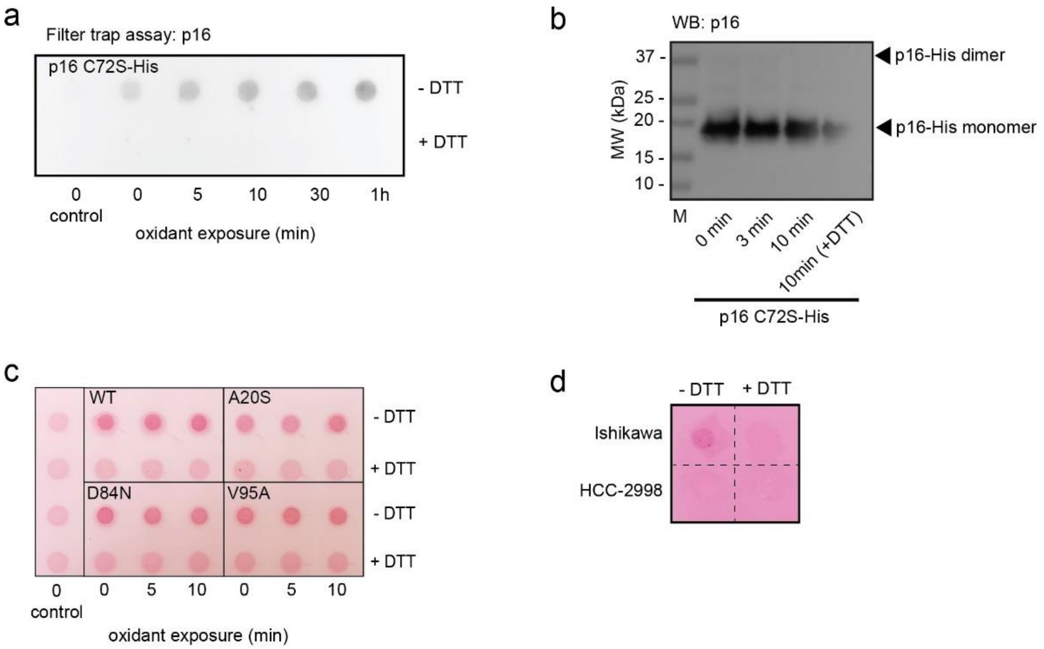

Supplementary Fig. 9: (**a**) Filter trap assay of p16 C72S-His; these samples were processed and measured on the same membrane and alongside wild-type p16 reported in Figure 7a. (**b**) WB for p16 C72S as described in Figure 7. (**c**, **d**) Ponceau S staining for the filter trap assays displayed in Figure 7c and 7d.
