# Supplementary material for: Amyloid formation and depolymerization of tumor suppressor p16INK4a are regulated by a thiol-dependent redox mechanism
Source: Nat Commun. 2024 Jul 1;15:5535. doi: 10.1038/s41467-024-49581-7 (PMC11217399; doi:10.1038/s41467-024-49581-7)
Supplement: Supplementary file 2 — Reporting Summary [file 41467_2024_49581_MOESM2_ESM.pdf]

nature portfolio

Corresponding author(s): Vanessa K. Morris, Christoph Göbl

Last updated by author(s): Jun 4, 2024

Reporting Summary

Nature Portfolio wishes to improve the reproducibility of the work that we publish. This form provides structure for consistency and transparency in reporting. For further information on Nature Portfolio policies, see our Editorial Policies and the Editorial Policy Checklist.

Statistics

For all statistical analyses, confirm that the following items are present in the figure legend, table legend, main text, or Methods section.

n/a	Confirmed	
☐	☒	The exact sample size (n) for each experimental group/condition, given as a discrete number and unit of measurement
☐	☒	A statement on whether measurements were taken from distinct samples or whether the same sample was measured repeatedly
☒	☐	The statistical test(s) used AND whether they are one- or two-sided<br>*Only common tests should be described solely by name; describe more complex techniques in the Methods section.*
☒	☐	A description of all covariates tested
☒	☐	A description of any assumptions or corrections, such as tests of normality and adjustment for multiple comparisons
☐	☒	A full description of the statistical parameters including central tendency (e.g. means) or other basic estimates (e.g. regression coefficient) AND variation (e.g. standard deviation) or associated estimates of uncertainty (e.g. confidence intervals)
☒	☐	For null hypothesis testing, the test statistic (e.g. F, t, r) with confidence intervals, effect sizes, degrees of freedom and P value noted<br>*Give P values as exact values whenever suitable.*
☒	☐	For Bayesian analysis, information on the choice of priors and Markov chain Monte Carlo settings
☒	☐	For hierarchical and complex designs, identification of the appropriate level for tests and full reporting of outcomes
☒	☐	Estimates of effect sizes (e.g. Cohen's d, Pearson's r), indicating how they were calculated

Our web collection on statistics for biologists contains articles on many of the points above.

Software and code

Policy information about availability of computer code

Data collection	Unicorn 7 (Cytiva) for size exclusion chromatography, SoftMax Pro for Thioflavin-T data, nanodrop 8000 spectrophotometer for protein concentrations, UVItec Q9 Advanced Chemidoc for western blotting, Jasco Spectra Manager for circular dichroism, QuantStudio 3 for dynamic scanning fluorimetry, ImageJ for electron micrographs, Thermo Xcalibur 2.2. SP1.48 for mass spectrometry, Cary eclipse kinetics applications version 1.1(133) for intrinsic fluorescence measurements
Data analysis	Amylofit webserver (Meisl et al, Nature Protocols 11, 252–272, 2016), Protein Thermal Shift Software 1.4 for melting temperature analysis, Thermo Xcalibur 2.2. SP1.48 for mass spectrometry, Pymol 2.5.0 for structural representation, Adobe Illustrator CS6, GraphPad Prism 10.2.3

For manuscripts utilizing custom algorithms or software that are central to the research but not yet described in published literature, software must be made available to editors and reviewers. We strongly encourage code deposition in a community repository (e.g. GitHub). See the Nature Portfolio guidelines for submitting code & software for further information.

Data

Policy information about availability of data

All manuscripts must include a data availability statement. This statement should provide the following information, where applicable:
- Accession codes, unique identifiers, or web links for publicly available datasets
- A description of any restrictions on data availability
- For clinical datasets or third party data, please ensure that the statement adheres to our policy

Data and materials availability: All data are available in the main text, supplementary materials, or as source data files. PDB structure files referred to are available:

2A5E [https://doi.org/10.2210/pdb2A5E/pdb], 1BI7 [https://doi.org/10.2210/pdb1BI7/pdb]. Mass spectrometry data has been deposited to FigShare [DOI: 10.6084/m9.figshare.25920136]. Source data has been deposited to FigShare [DOI: 10.6084/m9.figshare.25970758].

Research involving human participants, their data, or biological material

Policy information about studies with human participants or human data. See also policy information about sex, gender (identity/presentation), and sexual orientation and race, ethnicity and racism.

Reporting on sex and gender	n/a
Reporting on race, ethnicity, or other socially relevant groupings	n/a
Population characteristics	n/a
Recruitment	n/a
Ethics oversight	n/a

Note that full information on the approval of the study protocol must also be provided in the manuscript.

Field-specific reporting

Please select the one below that is the best fit for your research. If you are not sure, read the appropriate sections before making your selection.

- [x] Life sciences
- [ ] Behavioural & social sciences
- [ ] Ecological, evolutionary & environmental sciences

For a reference copy of the document with all sections, see nature.com/documents/nr-reporting-summary-flat.pdf

Life sciences study design

All studies must disclose on these points even when the disclosure is negative.

Sample size	For the various biophysical measurements performed, between two and ten repeats were acquired. These were sufficient due to the high precision of the measurement and the low biological variability observed.
Data exclusions	All data is presented in the manuscript and no data was excluded for analysis.
Replication	All experimental outcomes were able to be replicated.
Randomization	No randomization was necessary for this study.
Blinding	The biophysical methods applied do not require blinding because of the objective and automated data collection.

Reporting for specific materials, systems and methods

We require information from authors about some types of materials, experimental systems and methods used in many studies. Here, indicate whether each material, system or method listed is relevant to your study. If you are not sure if a list item applies to your research, read the appropriate section before selecting a response.

Materials & experimental systems

n/a	Involved in the study	
☐	☒	Antibodies
☐	☒	Eukaryotic cell lines
☒	☐	Palaeontology and archaeology
☒	☐	Animals and other organisms
☒	☐	Clinical data
☒	☐	Dual use research of concern
☒	☐	Plants

Methods

n/a	Involved in the study	
☒	☐	ChIP-seq
☒	☐	Flow cytometry
☒	☐	MRI-based neuroimaging

Antibodies

Antibodies used	All antibodies are commercially available.<br><br>anti-p16 monoclonal antibody (ab108349)

	HRP-conjugated goat anti-rabbit antibody (Dako, Agilent)<br>anti-phospho-Rb (Ser807/811) (Cell Signaling #9308)<br>anti-beta-actin (Sigma A5310)<br>anti-his-tag (Abcam ab18184)<br>anti-GST-tag (Abcam ab36415)<br>goat anti-mouse (DAKO P0447)<br>goat anti-rabbit (DAKO P0448)
Validation	anti-p16 monoclonal antibody (ab108349) was confirmed by western blotting using recombinantly produced p16 protein and was previously validated in IP, WB, Flow Cyt (Intra), IHC-P and tested in Human samples. Cited in 114 publications.<br><br>Phospho-Rb (Ser807/811) Antibody from Cell Signaling detects endogenous levels of Rb when phosphorylated at serine 807/811. The antibody may cross-react with Rb phosphorylated at Ser608. Cited in 493 publications.<br><br>anti-beta-actin (Sigma A5310) recognizes an epitope located on the N-terminal end of the β-isoform of actin. It specifically labels β-actin in a wide variety of tissues and species. Sigma provided citations with example western blots.<br><br>anti-his-tag (Abcam ab18184) recognizes His-tagged recombinant proteins or His-tagged proteins overexpressed in cells. ab18184 reacts to recombinant proteins containing the 6X His tag® or 10X His tag® fused to either the amino or carboxy terminus. Species reactivity: species independent. Previously validated in IP, ELISA, Dot, WB, ICC/IF and tested in Tag samples. Cited in 237 publications.<br><br>ab36415 is a mouse monoclonal antibody with high affinity to the glutathione-S-transferase (GST) from Schistosoma japonicum.This purified antibody will detect the expressed GST protein or a GST-fusion protein, and can be utilized for immuno-purification of GST-fusion proteins. Species reactivity: species independent. Previously validated in IP, WB and tested in Tag samples. Cited in 3 publications.

Eukaryotic cell lines

Policy information about cell lines and Sex and Gender in Research

Cell line source(s)	Source of each cell line: HCC-2998 from the NCI-60 panel (NCI-Frederick Cancer DCTD Tumor/Cell Line Repository), Ishikawa cells were purchased from ATCC, HEK293 were gifted from Prof. Mark Hampton
Authentication	Cell line authentication was performed with Applied Biosystems AmpFℓSTR® Identifiler™ for 15 STR loci.
Mycoplasma contamination	All cell lines tested negative for mycoplasma contamination.
Commonly misidentified lines<br>(See ICLAC register)	None

Plants

Seed stocks	*Report on the source of all seed stocks or other plant material used. If applicable, state the seed stock centre and catalogue number. If plant specimens were collected from the field, describe the collection location, date and sampling procedures.*
Novel plant genotypes	*Describe the methods by which all novel plant genotypes were produced. This includes those generated by transgenic approaches, gene editing, chemical/radiation-based mutagenesis and hybridization. For transgenic lines, describe the transformation method, the number of independent lines analyzed and the generation upon which experiments were performed. For gene-edited lines, describe the editor used, the endogenous sequence targeted for editing, the targeting guide RNA sequence (if applicable) and how the editor was applied.*
Authentication	*Describe any authentication procedures for each seed stock used or novel genotype generated. Describe any experiments used to assess the effect of a mutation and, where applicable, how potential secondary effects (e.g. second site T-DNA insertions, mosiacism, off-target gene editing) were examined.*
